# National Institute of Neurological Disorders and Stroke Clinical Trials Methodology Course: Summary and Accomplishments 2014–2023

**DOI:** 10.1212/NE9.0000000000200174

**Published:** 2024-11-08

**Authors:** Mark Quigg, Laurie Gutmann, Robin A. Conwit, Christopher S. Coffey, Roger J. Lewis, Courtney Miller, William J. Meurer

**Affiliations:** From the Department of Neurology (M.Q.), University of Virginia, Charlottesville; Department of Neurology (L.G., R.A.C.), Indiana University, Indianapolis; Department of Biostatistics (C.S.C.), University of Iowa, Iowa City; The Lunquist Institute (R.J.L.), Torrance, CA; and Departments of Emergency Medicine (C.M., W.J.M.), and Neurology (W.J.M.), University of Michigan, Ann Arbor.

## Abstract

The Clinical Trials Methodology Course (CTMC), given from 2014 to 2023, was conducted to educate early-career clinical investigators from various backgrounds in neurosciences in the design of clinical trials and to provide mentorship to enhance academic careers and retention plus improve research productivity and the likelihood of successful grant applications. This summary describes the rationale, history, structure, and trainee outcomes of the CTMC. The course used small groups, consisting of 1–2 clinical faculty advisor(s), 1 faculty biostatistician, and 2–4 trainees who met remotely approximately weekly over 12 weeks. Faculty and trainees then met for a 4-day in-person residential course. Follow-up activities included 2–3 follow-up remote meetings and a mock study section review of draft grant applications. The CTMC enrolled 243 trainees from 2014 to 2023 (excluding 2020) into Foundation (173) or other (70) tracks. Ninety-six percent of trainees remained in academic positions. Trainees published 7,666 peer-reviewed articles from their enrollment year to 2023 (mean 31.5 articles per trainee, or mean ± SD of 5.0 ± 5.1 articles per year per trainee). There were 7,120 unique articles; trainees were coauthors in 546. Of 173 Foundation Track trainees, 109 (63%) submitted an NIH grant as principal investigator or co-principal investigator, and 68 (62% of 109 submitters) were funded within a median of 3 years after course completion. Of the 243 total trainees, 91 (38%) were principal investigators for at least 1 NIH grant since their course participation to 2023. Trainees have participated as medical monitors, members of data and safety monitoring boards, investigators for NIH research networks, and faculty in the CTMC itself. CTMC has provided a robust foundation in clinical trial methodology in neuroscience research to a generation of clinical investigators.

## Introduction

The Clinical Trials Methodology Course (CTMC), supported by the National Institute of Neurological Disorders and Stroke (NINDS) of the NIH, was a longitudinal educational and professional development program conducted to educate early-career clinical investigators in neurosciences (neurology, neurosurgery, neuropsychology, emergency medicine, biostatistics) in the design of clinical trials and to provide mentorship with the goals of academic retention, enhancing research productivity, and improving the likelihood of successful grant applications. A key motivation for the trial was the need to increase capacity within the academic neuroscience community in the design and conduct of clinical trials. This summary describes the rationale, history, structure, and trainee outcomes of the CTMC.

## Rationale for a Course in Clinical Trial Methodology

Neurologic disorders result in an extraordinary burden on patients, caregivers, health care system, and society; the cumulative direct costs are $993 billion per year adjusted to current dollars.^1^ Improving the treatment of neurologic diseases requires clinical research, but research is limited, in part, by a shortage of capable, well-trained physician-scientists.^2^

Nearly a decade ago, the NIH established the Physician-Scientist Workforce Working Group to review the state of physician-scientists in medical research.^2^ The panel noted that the number of physician-scientists had declined, that the median age entering the workforce had increased, and that retirements had the potential to exceed replacements. The panel noted additional challenges as the requirements for clinical training for physicians had increased, the pressures to increase clinical productivity had risen, and retention of physicians in academic centers was falling. The coronavirus disease 2019 (COVID-19) pandemic is likely to have worsened critical shortages all along the clinical research pipeline because the “Great Resignation” caused experienced personnel in all categories, from physician-scientists to study coordinators, to leave clinical research.^3^ In this context, there is an urgent need to identify, develop, and nurture the next generation of physician-scientists capable of performing and leading critical clinical investigations in the treatment of neurologic illness and injury.

## History of the CTMC

A prior version of a course focused on clinical trials methodology was designed by personnel at NINDS and administered by investigators at the University of Rochester from 2006 to 2012. Often called “the Vail Course,” most activities of the course occurred in an extended retreat held in Vail, CO. The Vail Course was viewed as successful by NINDS personnel, but concerns existed about the length of the course (7–10 days); the expense of the location; and the limits of what could be accomplished in a single, intensive, but limited time period.

After conclusion of that program, NINDS program director Robin Conwit developed an R25 funding program with an open request for applications, and a grant supporting the CTMC described here was awarded to multiple principal investigators William J. Meurer (University of Michigan), Laurie Gutmann (University of Iowa), Chris Coffey (University of Iowa), and Roger J. Lewis (University of California, Los Angeles, and the Lundquist Institute at Harbor-UCLA Medical Center). The course was conducted for 9 years from 2014 to 2023 (with 1 year offering webinars only during the first wave of the COVID-19 pandemic). In its last years, the grant was administered and supported through the NINDS-Network for Excellence in Neuroscience Clinical Trials network (NeuroNEXT^4^). A final course was conducted in Spring 2024, supported by carry-over funds, but the follow-up of those recent trainees is not included here.

## Course Structure

The CTMC was held annually. Applications were submitted in January, and trainees were selected in February. The bulk of activities occurred through the spring and summer, with follow-up activities in the early fall. The CTMC structure focused on educational and mentoring activities conducted in small groups consisting of 2–4 trainees paired with a senior faculty member with experience in clinical trials investigations, a junior faculty clinical investigator, and a biostatistician. In the first year of the course, there was only 1 clinical faculty member and 1 biostatistical faculty member in each small group (adjusted in subsequent years). Before the residential portion of the course, the small groups met weekly through video conference, over approximately 6–12 weeks. This structure was used to address 1 concern regarding the Vail Course, namely the challenge of compressing virtually all course-related work into a single relatively short time interval. In addition, webinars developed by the core faculty were provided to all trainees every 2 weeks, providing didactics in aspects of clinical investigation. The webinars were advertised broadly and were open publicly. Beginning in 2021, the webinar programming was offered to fellows of NIH-funded clinical research networks as well. The topics and goals of weekly small group meetings and webinars were designed to reinforce each other and were structured toward the goal of building a complete clinical trial protocol and writing key elements of a grant proposal by the course's end (Table 1).

**Table 1:** Representative Curricula of Small Group and Webinar Topics for the CTMC

Small group topics	Webinar topics
Introductions and specific aims	Designing meaningful early phase clinical trials
Overall trial concept	Specific aims
Sections of protocol	What to know about sample size
Interventions and allocations	NINDS health disparities & global health agenda/common data elements
Primary and secondary end points and data management	Open forum Q&A and pre-residential course information
Budget	From statistical significance to clinical significance: time to dethrone or get rid of significance?
Adverse events and data and safety monitoring	Ethical issues in acute and chronic neurological conditions
Final statistical analysis strategy	What will the CTMC look like next year?

Abbreviations: CTMC = Clinical Trials Methodology Course; NINDS = National Institute of Neurological Disorders and Stroke.

Although trainees could differ in their overall trajectories in protocol creation, outlines provided guidance in preparation for the residential course. Webinars are posted for public access on YouTube.^5^

A 4-day, in-person residential course, following the sequence of small group meetings, served several purposes. First, it allowed a period of intensive face-to-face instruction to accelerate progress toward the completion of trial protocols and elements of grant applications. Second, it created an opportunity to provide additional structured didactic presentations and panel discussions to cover areas of clinical trial design, implementation, and research training not already covered. Third, it facilitated networking and the cementing of professional connections.

The location of the residential course alternated between the University of Michigan in Ann Arbor, MI, and the University of Iowa in Iowa City, IA, settings that brought a welcome conviviality of small, college towns. The American Academy of Neurology (AAN) provided additional financial support for the in-person course. Portions of the in-person course were attended by member(s) of the AAN clinical research subcommittee. A follow-up series of small group video conferences after the residential course culminated in a mock study section for final critiques.

Other features of the course served to ensure quality and legacy building. Formal surveys and open forums for trainee feedback were used to help guide the planning for the following year's curriculum. To enhance recruiting and to build “a family” of long-lasting contacts, the CTMC hosted reunions at the AAN annual meetings. The AAN provided support for this function. The iterative process of protocol development over the 9-month course period, as well as the cumulative interinstitutional and interdisciplinary network building, represented unique aspects of the CTMC that were not available elsewhere.

## Faculty

An executive committee headed by the principal investigators oversaw conduct of the course, presented webinars, and created other teaching materials. Additional faculty for small group instruction were generally recruited from established NIH-funded research networks: StrokeNET,^6^ Strategies to Innovate Emergency Care Clinical Trials Network (SIREN),^7^ and NeuroNEXT. Executive committee members and small group faculty provided most of the formal programming during the residential course. However, the residential course also featured a variety of supplemental faculty speakers who provided additional didactic lectures and networking opportunities. Faculty included participation of key members of the NIH-NINDS, giving trainees access to program officers and members of the training group.

## Trainees

Trainees were selected for participation in several different educational tracks over the lifetime of the CTMC. The initial priority criteria for the primary “Foundation Track” generally included early-career to early-mid–career physicians who were faculty or contracted to become faculty at an academic medical center. Some more senior researchers with no prior interventional trial experience were included. NIH faculty investigators were also welcome to apply. Later in the course, these criteria evolved to also include neuropsychologists, psychologists, nursing faculty, and biostatisticians. Trainees were not restricted by location or institution. In addition to the core Foundation Track, different tracks of trainees and proposals were trialed over the period of the program. The “Biostatistics Track” included a biostatistician trainee who joined a small group for the duration of the course and worked with the faculty biostatistician to develop trial design and data analysis plans for the other trainees of the small group. A “Biomarker Track” involved early-phase descriptive or validation trials in biomarker development. An “Advanced Track” involving adaptive clinical trial design, multicenter trials, or midphase trainees with more complex projects was trialed to support large-impact clinical trials.

## Proposals and Selection

The core of the required course application materials consisted of a 2-page research summary that included specific aims and a protocol outline. A trainee candidate biosketch and a letter(s) from a mentor and department chair, documenting appropriate supervision and support, were also required. Applications were reviewed by the executive committee and graded on the standard NIH 9-point scoring system.

The CTMC focused on proposals of early-phase clinical trials (i.e., not phase III or IV) in humans with neurologic disorders intended for public or foundation funding. Criteria did not specify funding level but, considering the trainee level, mentored awards (K23 or AAN or other foundation equivalents) were common targets. Biomarker studies were permitted, provided that they were framed as clinical trials.

Recruiting was performed with support of AAN and through the NINDS clinical research networks. Annual presentations and Q&A sessions at the AAN annual meetings and annual CTMC gatherings were hosted by the AAN. Application and course information were sent to chairs of US accredited neurology programs. AAN Clinical Research Fellowship applicants, primary investigators, and fellows from NINDS clinical research networks (NeuroNEXT, StrokeNET, and SIREN) were also sent application and course information through the AAN and through their respective networks. Previous trainees, their mentors, and mentees of trainees formed a referral network.

## Data Collection

Surveys were sent to all trainees annually, beginning the year after their participation in the course. Using the completed survey data, all outcomes were tracked from the year of trainee enrollment to December 2023. Survey and other data included:

- Demographics: Demographics were obtained from trainee surveys.
- Academic retention: Retention status at the end of 2023 was evaluated by a web search starting at the originating institution and current practice identified. We defined an “academic medical center” as a medical center including a hospital that (1) provides patient care, (2) confers medical degrees with an accredited school, (3) is the principal site of education of both medical students and postgraduate medical trainees, and (4) conducts medical research with oversight of an institutional review board.^8^
- Publication productivity: To determine publication history, we queried the website pubmed.gov to construct a publication database containing peer-reviewed articles from the year of enrollment to December 2023. Letters to the editor, replies, corrections, and errata were deleted, and duplicate references were combined.
- Grant productivity: Two methods were used. The first was the aforementioned surveys. The second was the use of NIH Reporter (reporter.nih.gov) from the year of enrollment to December 2023.

## Outcomes

### Trainees and Demographics

From 2014 to 2023, 243 trainees in annual class sizes from 15 to 42 enrolled in the CTMC. The course was suspended in 2020 because of the COVID-19 pandemic, and no small groups or residential course occurred; an open webinar series was offered. The 2021 class was performed remotely, and 2021 trainees were offered residential participation during the 2022 residential session. Enrollment over the duration of CTMC included 218 Foundation Track trainees (89.7%), 13 Advanced Track trainees (5.3%), 4 Biomarker Track trainees (1.6%), and 8 Biostatistics Track trainees (3.3%).

Of the 218 Foundation Track trainees, 203 (93%) returned the CTMC survey for basic demographics. The most common age category was 31–35 years with a substantial range (≤30 [2, 0.9%], 31–35 [19, 8.3%], 36–40 [23, 10.1%], 41–45 [12, 5.3%], 46–50 [3, 1.3%], >50 [1, 0.4%], not disclosed [2, 0.9%]). One hundred seven (49%) identified as female. One hundred sixty (73%) identified as White, 41 (19%) identified as Asian, 6 (3%) as Black or African American, and 11 (5%) preferred not to disclose their race. Seventeen (8%) identified as Hispanic.

One hundred thirty-seven (63%) of the Foundation Track trainees were neurologists (including pediatric and neonatal neurologists); 20 (9%) emergency medicine; and the remainder neurosurgery, psychiatry, psychology, biostatistics, nursing, and other specialties. Assistant professors were the most common rank at enrollment (129, 71%), followed by associate professors and fellows (37, 20%). Professors, clinical instructors, residents, and others comprised the remaining 15 (8%).

### Trainee Home Institutions and Academic Retention

At the time of participation in the CTMC, trainees hailed from a broad geographic distribution of academic medical centers. The 243 trainees came from 72 individual centers. The 5 most frequently represented institutions (Harvard-affiliated network, University of Michigan, University of Iowa, University of Rochester, and University of California-San Francisco) accounted for 25% of the participants. Seven trainees came from the NIH and 1 trainee from the Bill and Melinda Gates Foundation.

Of the 243 CTMC trainees, 233 (96%) remained at an academic medical center. One of these retired. Of the 10 who left, 1 trainee left for the US Food and Drug Administration, 2 for private practice, and 8 for nonresearch affiliates of primary academic centers.

### Trainee Publication Productivity

The 243 trainees published 7,666 peer-reviewed articles from their enrollment year of CTMC through December 2023, an average of 31.5 articles per trainee. Trainees were first authors on 1,275 of these articles (17%), with an average of 5.2 first-author articles per trainee. There were 7,120 unique articles; therefore, trainees were co-authors in 546 articles. Participants published into 1,391 different journals. The top 10 journals (impact factor 2022) (*Neurology* [10.1], *Stroke* [10.2], *Journal of Stroke and Cerebrovascular Disease* [2.7], *Journal of Neurointerventional Surgery* [8.6], *Frontiers of Neurology* [3.4], *Neurocritical Care* [3.5], *JAMA Neurology* [29], *Resuscitation* [6.2], *Annals of Neurology* [11.2], and *Journal of Neurosurgery* [4.1]) accounted for 20% of publications. Of the 7,120 unique articles, 587 (8%) contained the topic of “randomized controlled trial.”

**Figure 1:**
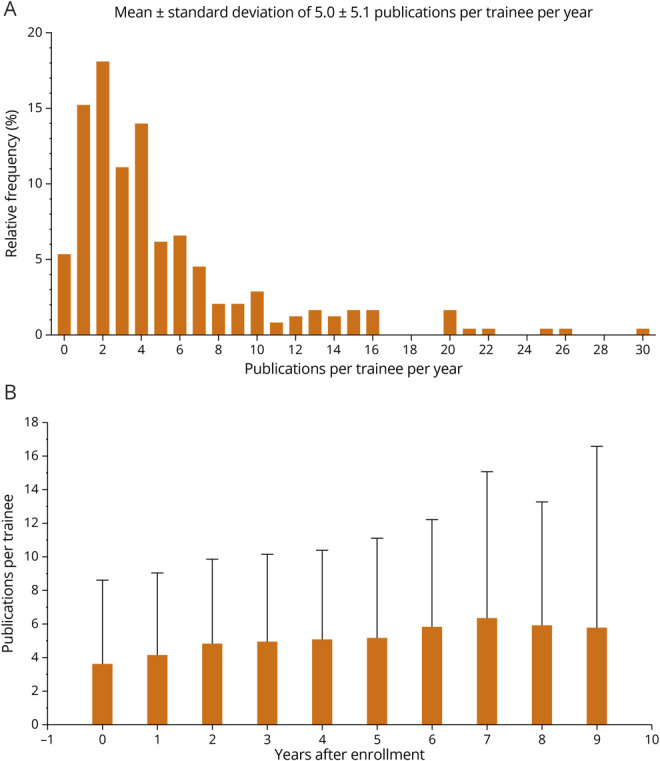
Peer-Reviewed Publications of 243 CTMC Trainees as Obtained From PubMed.gov (A) Frequency distribution of the per trainee annual number of peer-reviewed publications. (B) The mean and SD of the number of peer-reviewed publications per year, by the number of years from CTMC enrollment per trainee. Therefore, at 0 years, all 243 trainees are represented, and at year 9, only the initial Class of 2014 is represented. CTMC = Clinical Trials Methodology Course.

In adjusted annual productivity, trainees published a mean ± SD of 5.0 ± 5.1 articles per trainee per year after enrollment (Figure 1A). Participants were first author for a mean ± SD 0.22 ± 0.22 articles per participant per year. When plotted by years after enrollment in the CMTC (Figure 1B), the rate of publications per participant gradually increased, suggesting that scholarship after the CTMC was sustained and progressed over time.

### Grant Funding of Trainees

We used 2 sources of information regarding the receipt of grants. The first was trainee survey; the 15-member class of 2023 was not surveyed for this outcome. Of the remaining 203 Foundation trainees, 173 (85%) responded to requests for grant funding status. One hundred nine (63%) submitted an NIH grant as principal investigator or co-principal investigator, of which 68 (62% of submitters) were funded within a median time of 3 years after course completion. Seventy-five percent of trainees were funded either as principal investigator or as a site principal investigator in a multicenter trial. Trainees' roles on grants awarded ranged from serving as independent principal investigators to trainees on mentored awards. When reported, 53% of grants were R01/U01/U24/UH3/UF1, 27% K23/K02, and 20% R21/R34. NIH grant funding per year ranged from 43% to 84% of respondents (Table 2). Although we do not have a systematic count of funding from foundations or local sources, anecdotally we note that many of the trainees have been successful in securing such funding.

**Table 2:** Proportions of Those Trainees Per Year of Enrollment Successful in Acquiring NIH Funding Limited to Those Responding to At Least 1 Survey up to 2019

Year	Percentage
2014	55
2015	63
2016	68
2017	84
2018	43
2019	67

**Figure 2:**
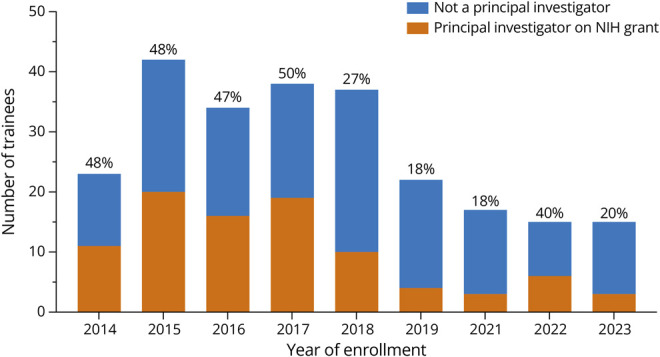
Proportion of Trainees by Year of Enrollment Who Were Awarded an NIH Grant Award as a Principal Investigator At Least Once From the Year of Enrollment to 2023 Source NIH Reporter (reporter.nih.gov).

The second source of information on grant funding was the NIH Reporter (reporter.nih.gov), in which all trainees were searched as a study principal investigator. Of the 243 trainees, 92 (38%) were principal investigators in an NIH-awarded grant at least once from the year of their enrollment to 2023. A plot of the proportion by year of enrollment shows that, in some course years, more than half of the trainees ultimately succeeded as a NIH-funded principal investigator (Figure 2). The proportion of NIH-funded trainees tended to rise with time, consistent with our expectation that early investment in trainees required time to pay off.

We note that at the time of the NIH Reporter query (April 2024), 6 more trainee applicants from enrollment years 2022 and 2023 were awarded their first NIH grants as principal investigators in the first quarter of 2024, bringing the total proportion of trainees who had become NIH-funded principal investigators to 40%.

### Other Trainee Benefits

Trainees reported that participation in the CTMC brought both direct and indirect benefits with respect to career development. Many of the trainees have become involved in NINDS networks, submitting projects that may have moved on to implementation, but also becoming involved in protocol working groups, medical monitoring, data and safety monitoring boards, and as principal investigators for network sites. Two trainees from early years served as faculty for small groups for more recent years of the CTMC. Each year, a former trainee returned to the in-person course to give a supplementary talk regarding their progress after the course.

Specific feedback, as documented in NIH progress reports from the CTMC, featured positive comments on learning and networking. For example, the CTMC created an “organized and structured way of infusing broad knowledge centered on rigor for the conduct of clinical trials—clear thoughtfulness (in) teaching broad aspects that will be helpful regardless of the trainee's field of study.” The interactions among faculty and trainees provided an “absolutely unique opportunity to gain feedback from key experienced investigators and reviewers, which gives an opportunity to identify which areas need further work.” Trainees said that during the course “hearing other people talk about their studies is extremely helpful,” and they found it valuable to participate in the trial design discussions for others in their group. Many described building up a network of long-term relationships, including those across different areas of research and neurologic disorders, and creating a support system for their efforts in trial design and development. The opportunity to meet senior academic faculty from a variety of institutions also helped trainees to identify sources of external mentorship and allowed access to high-ranking external faculty who can provide letters of support, an underrated but important career development tool. Trainee feedback also commented that the small group format allowed flexibility for clinical schedules, family commitments, and varying time zones.

### Faculty

The CTMC faculty team consisted of experienced mentors, clinician investigators, and biostatisticians devoted to developing and supporting neuroscience trials. The CTMC has had 43 course faculty participate as core faculty (small group leaders and residential course faculty) from 2014 to 2023. Of these, 39 faculty provided their demographic information.

Most of the faculty were aged between 41 and 60 years. Thirty-four (87%) faculty identified as White, 3 (8%) identified as Black or African American, 1 (5%) as Asian, and 1 (3%) nondisclosed. Eighteen (46%) identified as female. Nineteen (50%) of core faculty held the rank of Professor, 10 (26%) of Associate Professor, 3 (8%) of Assistant Professor, and 6 (16%) a nonacademic position. Twenty-four (62%) identified as a clinical researcher, 12 (31%) as biostatistician, 2 (5%) as federal government employee, and 1 (3%) as other. Faculty members' academic departments or divisions included neurology, emergency medicine, biostatistics, public health, neurosurgery, pediatric neurology, physical or occupational therapy, psychiatry, and radiology.

### Participation in Research Networks

Key collaborators of the CTMC included the NIH-funded research networks of StrokeNET, SIREN, and NeuroNEXT. StrokeNET, funded by the NINDS, undertakes both small and large studies to advance acute stroke treatment, stroke prevention, and recovery and rehabilitation after a stroke across the lifespan: The network consists of 27 regional centers across the United States. SIREN, funded by the NINDS, the National Heart Lung and Blood Institute, and the National Center for Advancing Translational Science, is organized to improve the outcomes of patients with neurologic, cardiac, respiratory, and hematologic emergencies by identifying effective treatments given in the earliest stages of care. SIREN consists of 11 regional hubs. NeuroNEXT focuses on the implementation of early and midphase clinical trials and biomarker validation in a variety of nonstroke neurologic disorders with an emphasis on recruitment for rare diseases. NeuroNEXT currently consists of 13 funded sites and 7 reserve sites. Cumulatively, these 3 networks have provided infrastructure to numerous projects providing access to cutting-edge research. We note that the faculty and participant intersections between the 3 networks and CTMC were mutually reinforcing.

CTMC participants with StrokeNet activities included 2 with StrokeNET protocol submissions and 1 achieving designation as a StrokeNET hub site principal investigator. CTMC trained 5 participants who were principal investigators for StrokeNet trials.

CTMC graduates with SIREN accomplishments included 1 trainee with a successful auxiliary trial from a SIREN trial, 1 SIREN hub principal investigator, 1 SIREN executive committee member/coinvestigator, and 2 principal investigators for 3 multisite SIREN or NINDS-funded trials.

In the NeuroNEXT program, 4 CTMC trainees submitted proposals to NeuroNEXT. A CTMC trainee has served as an independent medical monitor for a NeuroNEXT study, 2 others as a NeuroNEXT faculty member, and another as a biostatistics lead for the NeuroNEXT Data Coordinating Center.

### Limitations

A limitation of our report is that we lack a control group. The individuals enrolled in the CTMC, as evidenced by their appointments to a variety of respected institutions, represent a group who may have succeeded without participation. We acknowledge that a major limitation of any research training course is that identification and enrollment of any control group is fraught with logistical and logical shortcomings. We must rely on the testimony of the participants themselves, summarized above, who have stated that the CTMC was an important step in the development of their project and career. Another limitation was incomplete adherence to the series of trainee surveys.

### Future Directions

A new iteration of the CTMC was awarded in mid-August 2024 with plans to enroll classes from 2025 to 2030 headed by multiple principal investigators Mark Quigg (University of Virginia) and Laurie Gutmann (Indiana University). The new CTMC, based on the robust and “battle-tested” structure reported here, features many new endeavors with the following highlights. The first is to include a director of diversity, equity, and inclusion to promote the overall societal impact of trainees and proposals. The second is to include in the executive structure representatives from NINDS/NIH-funded networks (NeuroNEXT, StrokeNET, SIREN) and to seek trainees who intend to develop network-ready proposals to leverage resources in proposal development. A third is to introduce data science approaches in clinical investigation, leveraged through the new School of Data Science at the University of Virginia. We anticipate that adjustments to our follow-up database and surveys will further detail outcomes for future reference.

## Summary

Overall, the CTMC provided a valuable service to academically oriented clinician investigators in clinical neuroscience research and scholarship planning research careers involving clinical trials. The overwhelming majority of trainees remain in academic careers. Publications from the cohort, often involving information about randomized clinical trials, were widespread, impactful, and enduring. Trainees wrote grants, often successfully, and performed clinical trials and studies. Trainees took part in the academic and research enterprise comprising the infrastructure of systems funded by the NIH. Although we do not have a comparison group of early-stage academicians who did not participate in the CTMC, the success rate of retention and timing of grant submissions and funding is impressive. We anticipate that CTMC graduates will continue to fulfill leadership roles in future years. Finally, the networking opportunities cannot be quantified but were a key part of the CTMC experience. The CTMC has been an effective and consistent tool to provide physician-scientists with the knowledge and skills to succeed in a challenging medical research and economic landscape.

## Appendix Authors

**Table:**

Name	Location	Contribution
Mark Quigg, MD, MSc	Department of Neurology, University of Virginia, Charlottesville	Drafting/revision of the manuscript for content, including medical writing for content; major role in the acquisition of data; study concept or design; analysis or interpretation of data
Laurie Gutmann, MD	Department of Neurology, Indiana University, Indianapolis	Drafting/revision of the manuscript for content, including medical writing for content; major role in the acquisition of data; study concept or design; analysis or interpretation of data
Robin A. Conwit, MD	Department of Neurology, Indiana University, Indianapolis	Drafting/revision of the manuscript for content, including medical writing for content; major role in the acquisition of data; study concept or design; analysis or interpretation of data
Christopher S. Coffey, PhD	Department of Biostatistics, University of Iowa, Iowa City	Drafting/revision of the manuscript for content, including medical writing for content; major role in the acquisition of data; study concept or design; analysis or interpretation of data
Roger J. Lewis, MD, PhD	The Lunquist Institute, Torrance, CA	Drafting/revision of the manuscript for content, including medical writing for content; study concept or design; analysis or interpretation of data
Courtney Miller	Department of Emergency Medicine, University of Michigan, Ann Arbor, MI	Major role in the acquisition of data
William J. Meurer, MD, MS	Department of Emergency Medicine, and Department of Neurology, University of Michigan, Ann Arbor	Drafting/revision of the manuscript for content, including medical writing for content; major role in the acquisition of data; study concept or design; analysis or interpretation of data

## Glossary

AAN: American Academy of Neurology
COVID-19: coronavirus disease 2019
CTMC: Clinical Trials Methodology Course
NeuroNEXT: Network for Excellence in Neuroscience Clinical Trials network
NINDS: National Institute of Neurological Disorders and Stroke
SIREN: Strategies to Innovate Emergency Care Clinical Trials Network
